# Risk of eating disorders in a non-western setting: an exploratory study in Khartoum state, Sudan

**DOI:** 10.1007/s40519-016-0311-7

**Published:** 2016-09-01

**Authors:** Charlotte C. L. Lau, Elena Ambrosino

**Affiliations:** 1 Faculty of Health, Medicine and Life Sciences, University of Maastricht, Universiteitssingel 60, 6229 ER Maastricht, The Netherlands; 2 Department of Genetics and Cell Biology, Faculty of Health, Medicine and Life Sciences, Institute of Public Health Genomics, Research Institute GROW, University of Maastricht, Universiteitssingel 50, 6229 ER Maastricht, The Netherlands

**Keywords:** Eating disorder behaviors, Non-western setting, Women, Sudan, Determinants, Culture

## Abstract

**Purpose:**

Recent research suggests an emergence of eating disorders [ED] in non-western settings for unknown reasons. This research investigates the presence of ED in Khartoum State [Sudan], and explores relevant factors amongst women at risk of ED and stakeholders involved with mental health care and policy-making.

**Methods:**

Women from four summer schools were approached and screened for risk of ED using a validated and adapted form of the Eating Attitudes Test-26. Focus groups were performed within the schools, selected participants at high risk were interviewed, and interviews with stakeholders were performed.

**Results:**

Around a third (32.6%) of participants scored as having high risk of ED. Interviews showed recurring themes determining eating attitudes including: intention, knowledge, environment and habit. Stakeholders’ opinions depended on whether they work directly with those affected by ED or in policy-making. The former advocated increased attention on ED, the latter did not. Overall, services for ED were lacking.

**Conclusions:**

A high presence of negative eating attitudes was found amongst screened participants with high risk of ED. Individual intention overrides all other determinants for abnormal eating. Moreover, evidence suggests that westernization may attribute to ED, supporting the view that ED are culturally bound. The differing stakeholders’ views, together with other data found in this study, allow a number of recommendations for increasing awareness and identification of ED in Sudan.

## Introduction

Mental health (MH) issues impose an increasing challenge upon health systems in both developed and developing settings [1]. Research into eating disorders (ED) in non-Western settings remains neglected, despite a number of studies in the last two decades suggesting the emergence of ED behaviors. The reasons behind this are yet to be fully explored.

Sudan, an Afro-Arab country, has one study on this subject. In 2012, Fath-Al-Alim and colleagues identified ED symptoms in 21.2 % of 340 male and female university students [2]. This prevalence is similar to that in other Arab countries [3–7], as well as Western ones [8–13]. However, with only one small study, Sudan is a non-Western country where research into ED attitudes is neglected.

Eating disorders, particularly anorexia nervosa, have been considered a western phenomenon [14]. The reasons behind this, as well, as behind the current emergence of ED behaviors in non-Western settings, are unclear. As such, culture is a possible determinant of ED behaviors, though its degree of influence is debated. Investigating ED behaviors in Sudan could contribute to this debate.

Unlike previous research in non-Western settings, an all-rounded perspective is presented in this paper by providing opinions on ED from various groups: those personally vulnerable to ED, those working directly in MH care, and those with authority to change state- and nation-wide policy regarding MH.

The aim of this study is: [1] to provide an idea of the occurrence of ED attitudes in Khartoum State; [2] to explore opinions on the determinants of ED [including culture] of women who are at high risk of ED; [3] to investigate opinions on ED and MH of stakeholders, that is those working in MH care and policy-making.

## Methods

### Participants and study design

Females are at risk of ED, particularly those aged 15–19 [2, 15, 16]. Due to the challenges of setting and timing, convenience sampling was used to select four female summer schools in Khartoum State. There, 164 females aged 15–19 were approached and asked to fill in a questionnaire. Twelve questionnaires were excluded due to incompletion, leaving 152 participants (92.7 % of approached women). Seventeen of those were also interviewed; at this point saturation for interviews was met. For each location, the school principal signed a consent form on behalf of the students following the local guidelines.

In addition, relevant stakeholders, meaning those involved in MH care and policy-making, were interviewed via snowball sampling; giving a selection of psychiatrists, health psychologists, academics, governmental officials, and WHO representatives. Snowball sampling is a method of purposive sampling where a few persons with the required characteristics are initially identified and interviewed [17]. Those will in turn identify others who qualify as interviewees, and so on. The selected stakeholders come from Ahfad University for Women (Omdurman), University of Medical Sciences and Technology (Khartoum), two MH hospitals (Bashar Hospital in Bahri, and Tigani El Mahi Hospital in Omdurman), the Khartoum State Ministry of Health, the Sudan Federal Ministry of Health, and the WHO Sudan.

### Data collection

Data were collected over 5 weeks with the help of female volunteers, to ensure privacy, comfort and to maximize trust [18]. Interpretation and translation were provided by volunteers from Ahfad University for Women and Khartoum University. Various methods of data collection were used to facilitate validation of the data (triangulation); including questionnaires, focus group discussions, and semi-structured interviews (each lasting between 20 and 60 min).

### Questionnaire

As in similar studies [2–7, 19], the Eating Attitudes Test 26 (EAT-26) was used to identify individuals at high risk of ED. Prior to data collection three steps were taken to ensure no major problems existed with the questionnaire for our study population [20]. First, expert advice was sought from the authors of EAT-26 and nutritionists from Ahfad University for Women regarding an adaptation of such test for our study. Second, a pre-test using an adapted form of the EAT-26 was conducted on a sample of ten girls, similar to the population under study, resulting in a number of revisions. Third, a final check on our version of the EAT-26 was conducted with colleagues at Ahfad University and with the authors of EAT-26. An Arabic version was prepared, to be used verbally, which was re-translated and confirmed as suitable by the authors of EAT-26. This produced the final EAT-26 dialectically appropriate for the study population. Finally, an English version was distributed, accompanied with verbal Arabic translation exactly as written, to allow complete understanding amongst participants and better analysis by English-speaking researchers. Questions from participants were answered by the translators. Overall, literacy rates are 71.9 % in Sudan for ages 15 and over [21].

### Interviews and focus groups

**Fig. 1:**
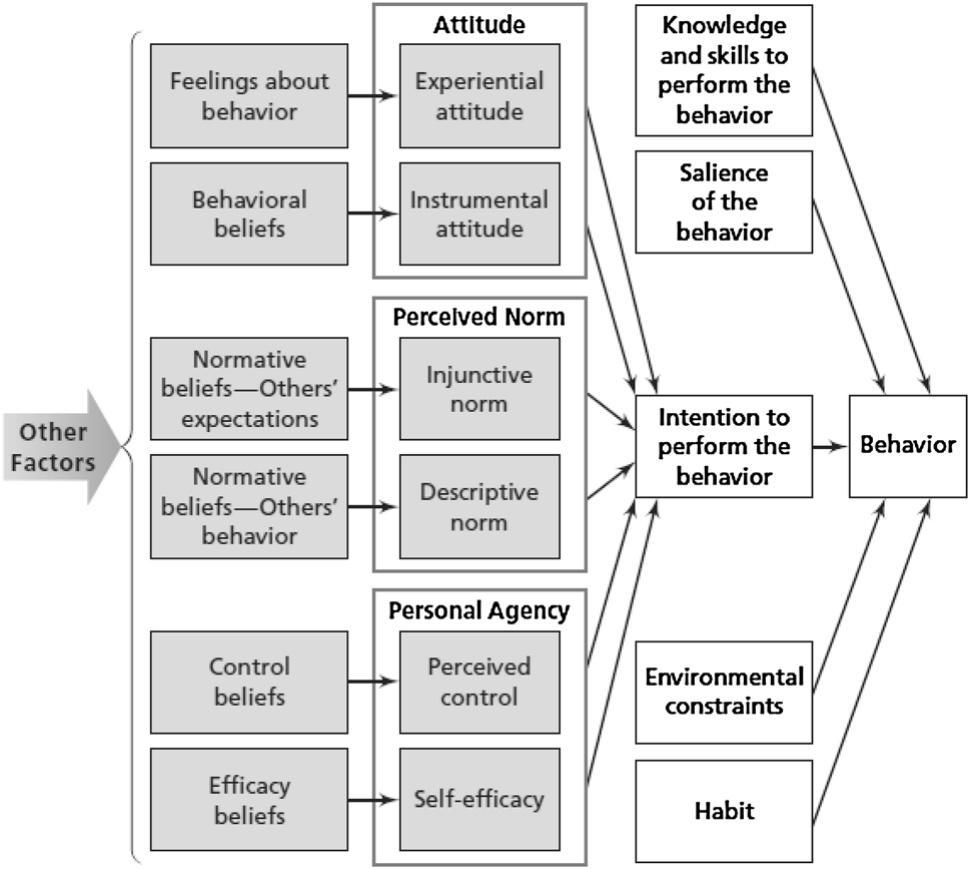
Integrated behavior model [22]

Four focus groups of 6–8 randomly selected women and 17 semi-structured interviews of women identified as having high risk for ED were conducted. The aim was exploring knowledge around ED and perceived determinants of ED behaviors. Determinants of ED behaviors were explored based on the integrated behavior model [22] represented diagrammatically in Fig. 1. It postulates that the most important determinant of behavior is intention. It identifies three constructs which contribute to intention: attitude, perceived norm and personal agency. Each of these three constructs has two main dimensions. Aside from intention, the integrated behavior model defines four other determinants that directly influence behavior including: knowledge and skills to perform the behavior; salience of the behavior; environmental constraints; and habit. Whilst other behavioral determinants are less important than intention, they are integral to determine whether behavioral intentions result in behavioral performance. The integrated behavior model may help to identify various determinants of ED behaviors in different populations, which can be of use for clinical decision making. In this study, semi-structured interviews explored intention, whilst focus groups explored the four other determinants.

Semi-structured interviews were also conducted with stakeholders and focused specifically on opinions regarding the burden of, knowledge of, and services available for ED and MH in Khartoum State and overall in Sudan.

English was used whenever possible, and interpreters helped where necessary. Interviews and focus groups were recorded, transcribed into verbatim, and Arabic was translated into English afterwards.

Where quotes have been used, ‘I’ represents the interviewer, ‘P’ represents the participant (followed by a number), and stakeholders are mentioned by their role. Anything originally stated in Arabic is marked with an asterix.

### Analyses

EAT-26 scores were calculated according to guidelines [23]. Interviews and focus groups were transcribed, and were analyzed verbatim with NVivo9, a software package used for qualitative data analysis [24]. Broad brush coding was conducted based on word frequency queries and text search queries, to identify prominent topics which occurred during interviews. This means words or phrases which were found to occur frequently or to be prominent throughout interviews were coded together based on topic. These topics were divided into broad subheadings provided by the integrated behavior model. Detailed analysis by re-reading all interviews provided fuller understanding of each individual’s experience with ED behaviors.

### Ethical approval

This research was conducted under the ethical approval obtained from Ahfad University for Women (AUFW). All procedures performed in studies involving human participants were in accordance with the ethical standards of the institutional and/or national research committee and with the 1964 Helsinki declaration and its later amendments or comparable ethical standards.

## Results

### Characteristics of the study population

Mean age of the sample was 15.6 years (SD 0.7), with mode age being 15 years, and a range between 15 and 19 years. Private school participants were 68.4 % of the sample and 31.6 % were from governmental schools. The selected schools are located in the three main cities in Khartoum State. The majority of participants (62.5 %) attended school in Omdurman, followed by Khartoum (26.3 %) and Bahri (11.2 %).

### Questionnaire results

The EAT-26 questionnaire was distributed to 164 participants and fully completed by 152 (92.7 %). Scores were analyzed according to official guidelines [23], and showed 47 participants (30.9 %) at high risk of ED.

### School interview and focus groups results

Of the 17 participants interviewed, all identified as at high risk of ED by EAT-26, 14 (82.4 %) described negative eating attitudes. Six (35.3 %) described extreme dieting, including regularly eating less than one meal per day or restriction from food until feeling dizzy or fainting; six (35.3 %) described binge eating; four (23.5 %) described both restriction and binge eating; seven (41.2 %) described crying about body weight or shape; one (5.9 %) described using pills for weight loss; one (5.9 %) described vomiting; and one (5.9 %) described excessive exercise. Four (23.5 %) described milder dieting. Three participants did not describe abnormal eating attitudes.

Semi-structured interviews explored intention whilst focus groups focused on other determinants. They were analyzed under the integrated behavior model [22]. The numbers of interviews and focus groups that discussed each determinant are shown in Tables 1 and 2.

**Table 1:** Results from semi-structured interviews: how often determinants of the integrated behavior model were discussed, by number and percentage over the total

Determinant	Occurrence of each determinant (%)
Intention	
Attitude	
Instrumental attitude	15 (88.2)
Experiential attitude	10 (58.8)
Perceived norm	
Descriptive norm	13 (76.5)
Injunctive norm	13 (76.5)
Personal agency	
Perceived control	12 (70.6)
Self-efficacy	2 (11.8)

**Table 2:** Results from focus groups: how often determinants of the integrated behavior model were discussed, by number and percentage over the total

Determinant	Occurrence of each determinant (%)
Knowledge and skills to perform the behaviour	4 (100.0)
Salience of the behaviour	4 (100.0)
Environmental constraints	3 (75.0)
Habit	3 (75.0)

Below is a detailed description of what emerged on the determinants of ED behaviors during the school interviews (for the determinant: intention) and focus groups (for the other determinants).

#### Intention

All interviews focused heavily on attitudes towards ED behavior. After having described the ED behaviors in which they partake, participants were asked about their behaviors’ expected outcomes (the instrumental attitude) and about their emotional responses to performing it (the experiential attitude). Instrumental attitudes were most prominently to look thinner, with 14 (82.4 %) participants describing this. Eight (47.1 %) mentioned to be more comfortable and confident; eight (47.1 %) to be pretty or beautiful, seven (41.2 %) to dress better or for style; five (29.4 %) to become happy; three (17.6 %) to improve health; one (5.9 %) to be free and unrestricted; and one (5.9 %) to avoid life problems. Experiential attitudes to ED behaviors were positive when desired behaviors were achieved, such as restricted consumption. Words used included “proud”, “comfortable”, and “happier”. Attitudes were negative towards failed or unwanted behaviors, such as binge eating or failed diets. Words included “guilty” and “scared of food”. Feelings of “confusion” and having both positive and negative experiential attitudes were also reported. For example, one participant (5.9 %) described feeling “tense and irritable”, whilst simultaneously happy, when abstaining from food.

The second construct of intention is perceived norm, comprising beliefs about what others are doing (descriptive norms) and beliefs about what others think oneself should be doing (injunctive norms). Participants believed that others strived for thinness and that others thought they should be thin too. Six (35.3 %) participants felt this came from friends, six (35.5 %) from family, three (17.6 %) from the community, two (11.8 %) from the internet, seven (41.2 %) from Western television and three (17.6 %) from Sudanese television. However, two (11.8 %) participants believed than Sudanese society found fuller women more attractive and three (17.6 %) participants identified a generational difference with mothers, fathers and aunties preferring fuller figures. Nonetheless, all participants followed an overriding personal desire for thinness, irrespective of the perceived norm.

The final construct of intention is personal agency, comprising perceived control and self-efficacy (confidence in performing a certain behavior). The latter occurred only briefly in two interviews, whilst perceived control was a common topic of discussion. Six (35.3 %) participants described poor control over food in regards to binge eating or failed or unwanted ED behaviors, and seven (41.2 %) described good control when successfully achieving desired ED behaviors such as food restriction.

#### The integrated behavior model’s other components of behavior

*Knowledge and skills* Poor understanding of ED was evident amongst all focus groups. When asked what ED are, one group (25 %) described being fat, obesity and diseases such as hypertension and diabetes. Two groups (50 %) discussed cleanliness of food and pollution in restaurants. Another group (25 %) described ED simply as the types of food you eat. Only one participant in the third focus group seemed to have a better understanding of the meaning of ED, describing “some psychological effect in the people so that they will think they are fat”.

*Salience* Two groups (50 %) believed ED are not salient in Sudan. The other two other groups (50 %) compared the salience of ED to that of poverty and starvation—they unanimously considered their struggles with poverty and starvation as more important than ED.

*Habit* ED pathology means ED behaviors are habitual, as sufferers lack control over their actions. Fourteen (82.4 %) described some habitual pattern of negative eating behavior.

*Environmental constraints* Three groups (75 %) focused their discussion on the lack of environmental constraints against ED behaviors. Family (often siblings and cousins), friends and community failed constraining ED behaviors and sometimes encouraged them through apathy, encouraging thinness, or participating in ED attitudes themselves. Television, internet and availability of weight-gaining food also encouraged ED attitudes. Contrarily, two groups (50 %) described one environmental constraint being the generational difference in beauty perception; with mothers, aunties and fathers preferring fuller figures as opposed to friends and siblings preferring thinner.

### Stakeholder interview results

Main themes occurring in stakeholder interviews included relevance of, knowledge about, services for, and future plans related to MH and ED.

#### Relevance of ED

An overwhelming majority, including the Minister of Health of Khartoum State, thought that ED were irrelevant for Sudan. However, psychiatrists and psychologists working directly with adolescents acknowledged a presence, though rare, and rise of ED in Sudan. Three had diagnosed various clinical or subclinical cases themselves. A 2012–2013 report conducted in all governmental primary schools by the Khartoum State Department of Maternal and Child Health found one child with ED. These same professionals discussed reasons behind the apparent emergence of ED in Sudan, including the rise of obesity. They also discussed the masking of ED by other disorders. One clinical psychologist described the complexity of eating problems in Sudan.

Health psychologist; Ahfad University for Women Trauma Centre:

… during our counselling I can’t say that we have encountered as many ED as we should expect. We are dealing with almost 8000 students, they range from 16 up to 26, or more for post-graduate studies…It seems that the aspect that is really worrying to them is more related to gaining weight, rather than loss of weight. So we have phenomena of people eating certain tablets to gain weight, which are mostly prescribed to diabetic patients, specifically in the lower proximities, like the legs – so it will fatten their legs, arms. So the culture is more embracing weight gain rather than weight loss, however we have noticed a shift in attitude regarding some of the girls who really want to go very thin and lose the weight…some girls just use, and that’s just from observation, or some of the cases I’m following, they use the starvation method, so they starve themselves basically. They don’t eat the whole day, so as to lose the weight, some of them reach the stage where they faint. However, are ED masked by depression? I would think so. There are several cases of depression masking an ED. We deal with it as only depression, and we look at the weight loss as a sign or symptom coupled with depression, not as a separate. So this is a tricky area, a grey area…However, the culture here in Sudan praise weight gain… they have a preference for a plump figure, rather than thin. However the new generation now there is shift, because they are exposed to, I would say western societies more, they want to fit in these tight jeans. To what extent this has now progressed to losing the weight through unhealthy habits, again this is not thoroughly researched, not to my knowledge at least…Through these [trauma] centers, we haven’t dealt, till now with a case that is suffering from bulimia, for example, or anorexia. However we do see students that look anorexic, and they do come with symptoms of depression, and now we deal with them… when you started to ask, I started to thinking… ‘uh huh, maybe this is, the depression is really masking the ED’.

#### Knowledge of ED

In general, it seemed that those stakeholders who demonstrated a good knowledge and understanding of ED were those who speculated its presence in Sudan. These individuals were concerned that other healthcare professionals had a poor knowledge of ED and might miss diagnosing them. A psychiatrist at Taha Baashar Hospital expressed her concern that doctors are more focused on physical morbidities, leaving MH issues ignored and neglected.

Child and adolescent psychiatrist; Taha Baashar Hospital:

I’m not very confident to say they [physicians] will think of ED. They will go as far as exploring every organic problem that could cause weight loss. And they might not at all think about ED… even with the child saying ‘I’m not eating’, they don’t think whether they are not eating willingly because they don’t want to be fat. I’m not sure of the awareness among doctors.

Another indication of poor knowledge of, or perhaps disinterest in, ED was that over half of the interviews (7 of 12) digressed to talking about MH in general despite attempting to discuss ED specifically.

#### Services for ED

MH services in Sudan are lacking. In Khartoum State Ministry of Health there is no MH department. On interviewing employees about work related to MH, responses were disjointed and often uncertain. Research found that two connections to MH exist, a liaison for feedback between the Curative Medicine Department and Taha Bashaar Hospital; and a School Health Program within the Primary Health Care Department.

In the Federal Ministry of Health there is a MH department. Two of its three employees were interviewed and complained of poor resources and lack of governmental interest. No protocol existed for ED management, and most healthcare workers did not know how to manage ED.

Few MH programs exist, and nothing specifically for ED. One program is the annual School Health Program, which performs health checks on all Khartoum State governmental primary schools, including a general MH development examination. Referrals are made to psychiatrists in MH clinics [which exist in five of the seven localities in Khartoum State]. As stated, only one child with an ED was identified in the 2012–2013 report.

The Ministry of Health was also in the process of implementing a project which began in 2012 to move tertiary carers into primary care centers, including psychiatrists.

Minister of health; Khartouom state Ministry of health:

“We are transforming many health centers… and moving psychiatrists there to go and see patients… But the antenatal physician, surgeon, they’re ahead of the psychiatrists. Psychiatrists are the last.”

A national problem is the poor health workforce. The 2012–2016 National MH Strategy states that “All or almost all (>80 %) psychiatrists emigrate from the country within 5 years of the completion of their training” [25].

#### Future plans related to MH and ED

Three governmental plans for MH were clear. First, the Acting Director of the Department of Mother and Child Health hoped to expand the School Health Program by creating MH clinics in two remaining localities. Second, the Director of Primary Health Care hoped to have 30–50 new doctors trained in psychiatry over the next 5 years. Thirdly, the Minister of Health of Khartoum State hoped to continue the plan of moving tertiary care, including psychiatry, into primary care centers. On interview, the Ministry of Health and WHO agreed that in light of other competing priorities and limited resources, this was an appropriate way to tackle MH in Khartoum State.

Contrarily, healthcare professionals believed more was needed in addition to the efforts above. This included rigorous scientific research to assess the magnitude of ED; increased ED awareness amongst the public, doctors and the government; and more resources in MH.

## Discussion

This study shows a high presence of negative ED attitudes amongst adolescent women in Khartoum State, at 32.6 %. Other non-Western studies have found figures between 11.4 and 40.5 % [2–7, 19]. Studies in Western countries have found figures between 11.5 and 25 % [8–13]. This provides support for observations that “the rates of ED in developing countries are fluctuating in a complex way and are rapidly outpacing those in industrialized countries” [4].

This study looked largely at the determinants behind ED behaviors, with intention being the most important. The strongest instrumental attitude behind intention was desire to be thinner despite the perceived norm. Factors contributing to this strong desire included: positive attitudes in achieving desired ED behaviors; negative thoughts around unwanted ED behaviors; perceived norms of thinness being attractive, and lack of control over ED behaviors. These align with typical features in ED pathology.

Other behavioral determinants encouraging ED behavior included lack of environmental constraints against ED behavior, the habitual nature of ED due to having its psychopathological roots in control [26], and poor knowledge of ED.

There was a perceived lack of salience for ED amongst Sudanese culture, particularly due to overriding concerns for poverty and hunger. Whilst this may initially appear to discourage ED attitudes, the Minnesota “Starvation Study” [27] showed that semi-starved individuals exhibit behaviors similar to those in ED, suggesting that ED behaviors may actually be the result of initial under-nutrition. Under-nutrition may thus be a determinant of ED behavior [28].

Historically ED, especially anorexia nervosa, have been considered to be culturally bound [14]. However, research in the 1980s revealed the emergence of ED in non-Western countries, debatably “transcending cultural boundaries” [3, 29]. Gorden [30] identified cultural characteristics of non-Western countries where ED have occurred; three of which are evident in Khartoum State. Firstly, global consumer culture is evident as Western media and beauty ideals influenced interview participants [2, 14]. Secondly, lifestyle changes associated with obesity were evident with participants consuming fattening foods and adopting sedentary lifestyles. Stakeholders also noted the rise in obesity. These changes are described in the nutrition transition [31], which contributes to the epidemiological transition where a shift from communicable to non-communicable disease (including MH issues) occurs, providing a possible explanation for the emergence of ED attitudes in Sudan. Thirdly, Gorden describes how paradoxical characters between the traditional and modern woman can create identity confusion [30]—that is the generational conflict between the idealization of fuller figures and Western thinner figures. ED behaviors have been described as a coping mechanism to deal with this [6]. These intrusions of Western ideals and lifestyle into Sudan suggest that westernization could attribute to ED, supporting the argument that ED are culturally bound, with the exception of under-nutrition driven ED behaviors.

The third part of the study revealed that most stakeholders thought ED were irrelevant—except those working directly with adolescents in MH. They suggested the perceived irrelevance of ED was either due to overwhelming subclinical amounts of ED or the masking of ED by other conditions like depression.

Healthcare professionals concerns included firstly insufficient knowledge and awareness of ED amongst the public, health professionals and the government. It is important that primary care physicians, as first point of contact, are adept in identifying and managing ED [32].

Overall, ED services and general MH services are lacking. On a governmental level this is evident by a small department comprising three employees in the Federal Ministry of Health, and its non-existence in Khartoum State. Moreover, in Khartoum State, ED detection could be strengthened if the School Health Program was expanded as planned by the government.

In the context of other prioritizations, the Ministry of Health and WHO agree that the country has made appropriate steps in tackling the burden of MH issues. However, these findings suggest the need for more effort on ED awareness and identification in Khartoum State.

### Limitations

Due to limited funding and barriers in accessing schools, convenience sampling and a small sample size were used, limiting generalizability. Indeed, the selected schools, and the fact that those were summer schools, may not offer an accurate representation of the general female population aged 15–19 in the state, and in the whole country of Sudan. Results found are for Khartoum State sample only; nonetheless can act as guidelines for future larger studies in Sudan offering a validated methodology and questionnaire in the local setting. Due to the study timeframe, summer schools rather than regular schools were approached which may have caused selection bias and affected sample representativeness. Translation between English and Arabic may have resulted in loss of meaning and detail throughout the study, though re-translations and assessment with the authors of the original EST-26 may have mostly prevented this problem.

## Conclusion

On exploring ED attitudes in Khartoum State, conclusions can be drawn from the three parts of this research. Firstly, a high risk of ED is apparent, which can only be confirmed through rigorous epidemiological research. Secondly, a number of possible determinants of ED attitudes have been identified; as well as possible explanations for its high presence. Lastly, diverging opinions between government and healthcare professionals indicate the need for improved communication regarding ED and other MH issues.

A number of recommendations for ED in Sudan can be made:

1. Improve communication and support between MH professionals and governmental bodies.
2. Conduct more research on the magnitude of ED.
3. Increase education and awareness amongst the public, healthcare professionals, and the government.
4. Expand the School Health Program as planned by the government.
